# Metainflammation, Mitochondrial Dysfunction, and Organokine Crosstalk: A Central Axis Linking Metabolic Syndrome to Cardiovascular Diseases

**DOI:** 10.3390/ijms27156554

**Published:** 2026-07-23

**Authors:** Ana Flávia Pontes Sodré, Lucca Gonsales Rodrigues, Kátia P. Sloan, Lance A. Sloan, Masaru Tanaka, Rui Curi, Larissa Naomi Takeda, Ricardo de Alvares Goulart, Ana Luiza Decanini Miranda de Souza, Claudia Rucco Penteado Detregiachi, Antonelly Cassio Alves Carvalho, Ricardo J. Tofano, Elen Landgraf Guiguer, Adriano Cressoni Araújo, Claudio J. Rubira, Anupam Bishayee, Vitor E. Valenti, Sandra Maria Barbalho

**Affiliations:** 1 Department of Biochemistry/Pharmacology, School of Medicine, University of Marília (UNIMAR), Avenida Higino Muzzi Filho 1001, Marília 17525-902, SP, Brazil; 2 Texas Institute for Kidney and Endocrine Disorders, Lufkin, TX 75904, USA; 3 Endocrinology Department, University of Texas Medical Branch, Galveston, TX 75904, USA; 4 HUN-REN-SZTE Neuroscience Research Group, Danube Neuroscience Research Laboratory, Hungarian Research Network, University of Szeged (HUN-REN-SZTE), 6725 Szeged, Hungary; 5 Butantan Institute, São Paulo 05508-040, SP, Brazil; 6 Postgraduate Program in Structural and Functional Interactions in Rehabilitation, University of Marília (UNIMAR), Marília 17525-900, SP, Brazil; 7 Department of Pharmacology, College of Osteopathic Medicine, Lake Erie College of Osteopathic Medicine, Bradenton, FL 34211, USA; 8 Systematic Reviews and Meta-Analyses Center, School of Philosophy and Sciences, São Paulo State University, Marília 17525-900, SP, Brazil; vitor.valenti@unesp.br; 9 UNIMAR Charity Hospital, University of Marília (UNIMAR), Marília 17525-902, SP, Brazil

**Keywords:** metainflammation, oxidative stress, mitochondrial dysfunction, MetS, cardiovascular diseases, organokines

## Abstract

Metabolic Syndrome (MetS) is a complex and multifactorial condition characterized by insulin resistance, visceral obesity, dyslipidemia, hypertension, and chronic low-grade inflammation, all of which contribute to increased cardiovascular risk. Central to its pathophysiology is metainflammation, a persistent inflammatory state closely linked to oxidative stress and mitochondrial dysfunction. This review aims to provide an integrated and updated overview of the interplay between metainflammation, oxidative stress, mitochondrial dysfunction, and organokine signaling in the development and progression of MetS and its cardiovascular complications. Current evidence indicates that mitochondrial dysfunction plays a pivotal role by promoting excessive production of reactive oxygen species (ROS), impairing ATP synthesis, and disrupting redox balance, thereby exacerbating insulin resistance and endothelial dysfunction. In parallel, dysregulated secretion of organokines—including adipokines, myokines, hepatokines, cardiokines, osteokines, and renokines—alters interorgan communication and amplifies pro-inflammatory and atherogenic pathways. Additionally, gut microbiota contributes to metabolic homeostasis through the production of short-chain fatty acids, whereas dysbiosis is associated with worsening metabolic parameters. Collectively, these interconnected mechanisms establish a self-perpetuating cycle that drives metabolic dysfunction and cardiovascular disease progression. This review highlights the central role of the metainflammation–mitochondrial dysfunction axis and emphasizes the importance of organokine-mediated crosstalk as a key regulator of systemic metabolism. Targeting these pathways may represent a promising strategy for the prevention and management of MetS and its associated complications.

## 1. Introduction

Metabolic Syndrome (MetS) is a global health concern that affects more than one in four adults worldwide [1]. It is often associated with industrialized countries, where people are sedentary and constantly eat fast food that is characterized by being composed mainly of high levels of saturated fat, carbohydrates, sugar, and lipids, affecting the health and quality of life [2,3,4,5].

MetS is characterized by metainflammation, a result of genetic predisposition combined with changes in the body, such as obesity, insulin resistance, elevated abdominal circumference, dysglycemia, and cardiovascular disease (CVD) which also affect the liver, brain, and intestine [6,7,8]. These processes, when combined, affected the white adipose tissue (WAT). This endocrine tissue is responsible for the secretion of adipokines (such as leptin, visfatin, and resistin), cytokines, and cells, including endothelial cells, macrophages, fibroblasts, and leukocytes. Chronic energy surplus promotes adipocyte hypertrophy and expansion of white adipose tissue (WAT), leading to profound alterations in its endocrine and secretory profile [9,10]. An example of this is the increase in leptin secretion, leading to hyperleptinemia, which can affect satiety. The lack of sensitivity to the hormone in the brain does not stop the hunger stimulus, even when people have already eaten enough [11,12].

**Figure 1:**
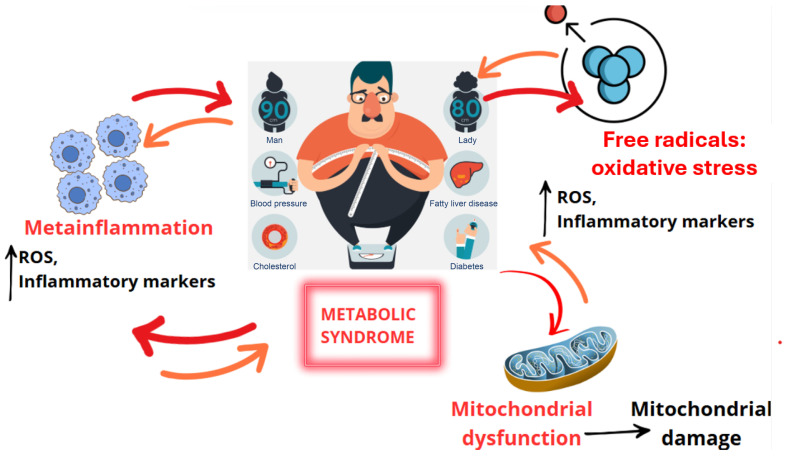
Central axis of MetS: metainflammation, oxidative stress, and mitochondrial dysfunction. Schematic representation of the central pathophysiological triad underlying Metabolic Syndrome. Metainflammation, oxidative stress, and mitochondrial dysfunction interact bidirectionally, forming a vicious cycle that perpetuates insulin resistance, endothelial dysfunction, and tissue injury. These interconnected processes contribute to the development and progression of cardiovascular disease and other metabolic complications. Each element of the triad amplifies the others, exacerbating systemic inflammation and impairing cellular energy homeostasis; ROS, Reactive Oxygen Species. Figure prepared with Canva.com.

Once the syndrome is established in the human body, there is an increase in pro-inflammatory adipokines produced by WAT, leading to chronic inflammation, increased secretion of pro-inflammatory cells, and decreased secretion of adiponectins. In this scenario, there is also an elevated production of reactive oxygen species (ROS), due to mitochondrial dysfunction, resulting in oxidative stress that can lead to irreversible systemic tissue damage [13,14]. The mitochondria are cellular organelles responsible for producing energy by generating adenosine triphosphate (ATP) through oxidative phosphorylation (OXPHOS). In normal conditions, this organelle produces ROS, but enzymes neutralize them before they can damage mitochondria and other organelles. However, due to insulin resistance, the organism in the MetS works differently, altering normal metabolic functions, causing mitochondrial dysfunction and, consequently, altering the electron transport chain, producing ROS that are no longer fully neutralized. In addition to this factor, mitochondrial dysfunction results in ATP deficiency, disrupting all cellular processes that require energy, and can lead to apoptosis [15,16]. Figure 1 summarizes the central axis of MetS: metainflammation, oxidative stress, and mitochondrial dysfunction.

The consequences of these changes in the body can lead to lower cognitive performance, vascular dementia, tissue fibrosis, atherogenesis, and other cardiovascular problems. For example, studies have shown that oxidative stress is linked to atrial fibrillation and can also contribute to neuroinflammation. Others have demonstrated a relationship between hepatic and pancreatic steatosis [17,18]. Regardless of location, mitochondrial dysfunction and oxidative stress induced by MetS result in systemic changes, altering the function of several organs and causing various diseases [19,20,21,22].

Although metainflammation, oxidative stress, mitochondrial dysfunction, organokine imbalance, and gut microbiota dysbiosis have each been implicated in MetS, these processes are often discussed as separate mechanisms. This fragmented view limits understanding of how local metabolic stress in adipose tissue, skeletal muscle, liver, heart, bone, kidney, and intestine becomes translated into systemic cardiometabolic injury. In particular, the integration of mitochondrial dysfunction with organokine-mediated interorgan crosstalk remains insufficiently synthesized, despite its potential relevance for biomarker discovery and multi-target therapeutic strategies.

Therefore, this review aims to synthesize current evidence on the metainflammation–mitochondrial dysfunction axis in MetS, with particular emphasis on organokine-mediated interorgan crosstalk, gut microbiota-derived metabolites, and their contribution to cardiovascular disease progression. By integrating these mechanisms, this review seeks to provide a systems-level framework for identifying convergent biomarkers and therapeutic targets in cardiometabolic disease.

### Literature Search Strategy

This narrative review was based on a comprehensive literature search conducted in PubMed/MEDLINE, EMBASE, and Google Scholar. The search included relevant articles published up to May 2026, with particular emphasis on studies published during the last 10 years.

The search strategy combined Medical Subject Headings (MeSH) terms and free-text keywords, including “metabolic syndrome,” “organokines,” “adipokines,” “myokines,” “hepatokines,” “cardiokines,” “osteokines,” “renokines,” “gut microbiota,” “dysbiosis,” “short-chain fatty acids,” “mitochondrial dysfunction,” “oxidative stress,” “metainflammation,” “insulin resistance,” “endothelial dysfunction,” “cardiovascular disease,” and “interorgan crosstalk.” Boolean operators (AND/OR) were applied to refine the search and retrieve the most relevant publications.

Studies were selected based on their relevance to the role of organokine-mediated interorgan communication, mitochondrial dysfunction, oxidative stress, inflammation, gut microbiota, and cardiometabolic complications in metabolic syndrome. Conference abstracts, editorials, and publications with limited relevance to the scope of this review were excluded.

As this is a narrative review, the objective of the literature search was not to perform a systematic assessment of the available evidence, but rather to identify, critically evaluate, and integrate the most relevant and up-to-date studies addressing organokine-mediated interorgan crosstalk and its contribution to the pathophysiology of metabolic syndrome and its cardiometabolic complications.

## 2. MetS and Metainflammation

MetS, also known as Syndrome X and VIRAS (visceral insulin resistance adiposity syndrome), is associated with obesity (primarily abdominal fat), insulin resistance, hypertension, elevated triglycerides, and reduced high-density lipoprotein (HDL-c) [23,24]. This scenario is more prevalent in adults, it is closely related to the risk for CVD, and it is a warning sign, since CVD are the leading cause of death in the world [2]. Studies have shown that obese individuals experience a significant increase in white adipose tissue throughout the body [25]. This change alters the integrity and sensitivity of cells to hormones such as insulin and adiponectin, contributing to metabolic dysfunction [26,27]. Besides WAT, there is also the brown adipose tissue (BAT) which plays distinct functions. The first type is characterized by cells containing a large unilocular lipid droplet that is associated with metabolism and fat storage and also serves an endocrine function [4]. These cells can be found in the viscera or subcutaneous tissue [28]. At the same time, the second is associated with the body’s thermal regulation, because its cells have a high mitochondrial density, enabling energy dissipation through uncoupled mitochondrial respiration that produces heat and maintains body temperature [29,30,31]. In patients with MetS, excess calorie consumption leads to an increase in the amount of visceral WAT. As a result, the WAT will be responsible for the release of hormones and pro-inflammatory cells that will cause imbalance in glucose metabolism, insulin resistance, liver complications, and increased release of free fatty acids (FFAs) and cholesterol, establishing a condition known as metainflammation [32,33,34,35].

Once MetS is established, profound metabolic disturbances develop across multiple organs. WAT undergoes marked endocrine remodeling characterized by adipocyte hypertrophy and increased infiltration of pro-inflammatory macrophages. This inflammatory microenvironment promotes the production of cytokines such as tumor necrosis factor-α (TNF-α) and interleukin-1β (IL-1β), while activating intracellular signaling pathways, including IκB kinase (IKK) and c-Jun N-terminal kinase (JNK), within adipocytes. Collectively, these alterations contribute to chronic metainflammation, insulin resistance, and the progression of metabolic dysfunction [36,37,38,39]. Macrophages (classified as M1 in this case) derived from monocytes leave the bloodstream and are responsible for triggering a series of inflammatory responses in adipose tissue. They release excess free FFA through changes in lipolysis which can activate JNK and IKKα. The IkB kinase mediators (IKKα and JNK), when activated, are responsible for the cascade release of pro-inflammatory mediators, in addition to causing insulin resistance [40]. Furthermore, interleukin-6 (IL-6) that is released by adipocytes and macrophages has been associated with endothelial dysfunction. After release, it stimulates the renin–angiotensin system to express more angiotensin II type 1 receptors (AT1) which are related to the development of atherosclerosis [41,42,43,44]. Furthermore, TNF-α release contributes to adipocyte hypertrophy and stimulates hepatic lipolysis leading to increased circulating FFA [45,46,47,48]. With all these changes, several vicious cycles are established; for example, insulin resistance is linked to the development of obesity, and obesity also leads to insulin resistance [49,50,51].

Normally, the binding of insulin to the receptor (IRS) leads to the phosphorylation of tyrosine kinase residues in the beta subunit, triggering a signaling cascade within the cell that culminates in the release of GLUT-4 (an insulin-sensitive glucose transporter) to the cell plasma membrane. During metainflammation, activation of IκB kinase α/β (IKKα/β) promotes serine/threonine phosphorylation of insulin receptor substrate (IRS) proteins, disrupting insulin receptor signaling and contributing to insulin resistance [52]. Simultaneously, adipocyte hypertrophy induces a marked shift in the adipose tissue secretome, characterized by increased production of pro-inflammatory adipokines and cytokines together with reduced secretion of metabolically protective mediators. These alterations are accompanied by dysregulated lipid and cholesterol metabolism, reinforcing chronic inflammation, mitochondrial dysfunction, and systemic metabolic impairment. This inflammatory response leads to an excessive increase in the release of pro-inflammatory adipokines [53,54,55]. In obese individuals, adipose tissue undergoes endocrine remodeling characterized by increased secretion of leptin (despite the development of leptin resistance), TNF-α, IL-6, and monocyte chemoattractant protein-1 (MCP-1), together with reduced production of adiponectin and omentin. Rather than isolated changes in individual adipokines, obesity is characterized by a disruption of the physiological balance between metabolically protective and pathological adipokines. This adipokine imbalance amplifies chronic low-grade inflammation, promotes insulin resistance, and perpetuates metainflammation [56,57]. Thus, mediators such as leptin acts on the hypothalamus to reduce hunger and are elevated in overweight individuals. However, due to the organism’s inflammatory state, this adipokine cannot efficiently perform its role, characterizing leptin resistance in individuals with MetS [58,59]. In contrast, adiponectin which facilitates greater glucose entry into cells by increasing insulin sensitivity is present in lower percentages in metainflammation. This factor further contributes to the development of type 2 diabetes mellitus (T2DM) in individuals with MetS [60].

Chronic low-grade inflammation disrupts interorgan communication, particularly along the gut–liver–adipose tissue axis, thereby contributing to metabolic dysfunction. In addition to the reciprocal interactions between adipocytes and hepatocytes described above, MetS is associated with impaired FGF21 signaling in adipose tissue. Although FGF21 is primarily secreted by the liver as an endocrine regulator of energy metabolism, obesity is frequently accompanied by FGF21 resistance, which reduces its ability to stimulate energy expenditure, fatty acid oxidation, and insulin sensitivity in adipose tissue. Consequently, these alterations further exacerbate adipose tissue dysfunction and systemic metabolic disturbances [61,62,63]. The mechanism of action of FGF21 involves binding to its receptor (FGFR1c), as well as its coreceptor, β-klotho, which is expressed only in metabolic tissues [64]. Studies indicate that, once bound to its receptor, FGF21 increases glucose uptake in WAT, thereby improving adipocyte thermogenesis and contributing to reduced insulin resistance and increased adiponectin secretion [65,66]. The reduction in its action during obesity, even with high liver production of this hormone, is due to receptor resistance to FGF21, which is caused by the individual’s inflammatory state. Consequently, changes in adipose cell metabolism lead to increased fat storage and, as a domino effect, resulting in weight gain [67,68].

All of these changes cause an imbalance in metabolism, leading to the release of mediators that, rather than reducing the inflammatory state, further increase it, impairing the organism’s overall functioning and creating a vicious cycle of inflammation and oxidative stress [69,70,71].

## 3. Oxidative Stress as an Amplifier of Metainflammation and Vascular Injury in MetS

As previously shown, in MetS, the sustained intake of calories contributes to the inflammatory process in WAT, leading to alterations in lipid metabolism. A series of studies has shown that, at the molecular level, mitochondria in WAT produce ROS, leading to mitochondrial dysfunction and lipid oxidation [24,72,73,74]. The mechanism is composed of a series of altered reactions due to a large amount of electrons being transferred to the respiratory chain (during energy production by mitochondria), resulting in an imbalance between antioxidant and oxidant species. The most serious consequence of this imbalance is that reactive oxygen species can interact with the cell’s DNA, altering protein function or disrupting signaling pathways, ultimately leading to programmed cell death [75,76,77].

In the inner mitochondrial membrane, during the mitochondrial electron transport chain (ETC), the NADH (nicotinamide adenine dinucleotide) molecule donates an electron to ubiquinone (CoQ), generating the reduced molecule ubiquinol (QH2) [78]. QH2 is oxidized by complex III, removing electrons from the molecule one at a time, which are transferred to cytochrome C. Then, this cytochrome carries these electrons to complex IV, and oxygen serves as the final electron acceptor, generating water. All these oxidation and reduction reactions create an electrochemical gradient across the membrane spaces. Ultimately, during the entry of H+ into the mitochondrial matrix, ADP is phosphorylated to ATP [79]. In metabolism, reactive oxygen species are produced in the mitochondria during the reduction of NADH to NAD+ in oxidative phosphorylation [80]. The NADPH (nicotine adenine dinucleotide phosphate) oxidase family of enzymes is extremely important in understanding the mechanism of oxidative stress, since the enzyme NADPH oxidase 2 (NOX2) (catalytic subunit of NADPH) is responsible for the overproduction of ROS [81,82,83].

When an individual consumes high-calorie foods or expends more energy than they can metabolize during the day, nutrients accumulate in the body. This excess of ingested nutrients causes an overload of oxidative phosphorylation [84]. This excess of ingested inputs for metabolism leads to an overload of oxidative phosphorylation [85]. Associated with this overload, individuals with MetS are constantly under metabolic stress, as a significant amount of NADH is released, and the body requires little ATP [86]. Since the caloric expenditure is small compared to the food intake, there is a disproportion between the amounts of NAPH and NAD+ [87,88,89]. All of this, when combined, generates an escape of electrons during their transfer between the complexes, reacting with oxygen and forming $0_{2}$^−^. Together, these changes promote electron leakage from the mitochondrial respiratory chain, allowing partially reduced oxygen species, particularly superoxide anion, to accumulate and initiate downstream oxidative damage [76,90,91].

Once produced, ROS directly promote an inflammatory environment, as they activate NF-κB (nuclear factor-κB) transcription, a complex involved in the immune response during inflammation [92,93,94]. This factor is responsible for activating signaling pathways that produce pro-inflammatory cytokines, including IL-6, TNF-α, and MCP-1, as well as IL-1β. These cytokines, in turn, attract additional pro-inflammatory cells, leading to angiogenesis and fibrosis. Furthermore, they can act on the cell nucleus, resulting in cellular dysfunction [95,96]. In addition, TNF-α inhibits insulin receptor signaling in cells, leading to increased insulin resistance and, in turn, enhanced lipolysis, the release of FFA into the circulation, and decreased glycogen synthesis [97,98,99]. All these processes are closely linked to the establishment of an environment characterized by chronic inflammation and oxidative stress, which, consequently, leads to MetS. The consequence is that normal metabolic reactions are affected and adipose tissue function is altered, leading to changes in systemic metabolism [96,100]. One of the major problems with this syndrome is that, once established, the release of pro-inflammatory factors attracts additional inflammatory cytokines, forming a vicious cycle [101,102].

High levels of ROS cause vascular endothelial dysfunction, as these molecules induce lipid peroxidation and protein oxidation and can even lead to changes in cellular DNA [103,104]. In addition, ROS can alter the expression of adhesion molecules, such as P- and E-selectins, on endothelial cells, leading to endothelial dysfunction [105,106]. These cellular changes induced by ROS damage the cell membrane, releasing damage-associated molecular patterns (DAMPs) and pathogen-associated molecular patterns (PAMPs), which activate inflammatory pathways [107]. As noted above, cells such as macrophages and neutrophils are recruited to the tissue, where they attempt to eliminate the aggressive agent by releasing cytokines such as TNF-α and IL-6, which, in turn, attract additional inflammatory cells, creating a constant inflammatory environment [108,109]. Still in the vascular bed, the homeostatic imbalance of ROS leads to the oxidation of low-density lipoprotein (LDL). Macrophages recognize the oxidized LDL (ox-LDL) through scavenger receptors as a foreign cell, and the macrophages phagocytose the ox-LDL, generating foam cells [110,111]. After that, with a dysfunctional endothelium, these foam cells adhere to the intimal layer of the vessel, initiating atherosclerotic plaque formation [112,113,114]. Over time, ROS excess causes the rupture of endothelial cell junctions. It increases vascular permeability, allowing foam cells to accumulate in the vessel and form an atherosclerotic plaque, which narrows the blood vessel lumen and hinders blood flow. If this occurs in the coronary artery, the obstruction of the vessel leads to infarction [115,116,117,118]. Oxidized low-density lipoprotein receptor-1 (LOX-1) binds ox-LDL, triggering endothelial activation and platelet aggregation. The interaction between activated platelets and the dysfunctional endothelium, together with ROS-induced activation of matrix metalloproteinases (MMPs), promotes degradation of the extracellular matrix and thinning of the fibrous cap of atherosclerotic plaques. As plate stability declines, the risk of plate rupture and subsequent thrombus formation markedly increases [119,120,121]. Once formed, this thrombus can travel through blood vessels, reaching the heart, where it can obstruct the coronary arteries and cause a heart attack [122,123].

Beyond metabolic regulation, organokine imbalance is increasingly recognized as a major contributor to vascular dysfunction. Altered secretion of adipokines, hepatokines, cardiokines, osteokines, and renokines promotes endothelial dysfunction by reducing nitric oxide (NO) bioavailability, enhancing oxidative stress, increasing endothelial permeability, and upregulating adhesion molecules such as VCAM-1 and ICAM-1. These changes facilitate leukocyte recruitment, vascular inflammation, and endothelial activation, representing early events in the development of cardiovascular complications associated with MetS.

## 4. Mitochondrial Dysfunction as a Bioenergetic Driver of MetS Progression

**Figure 2:**
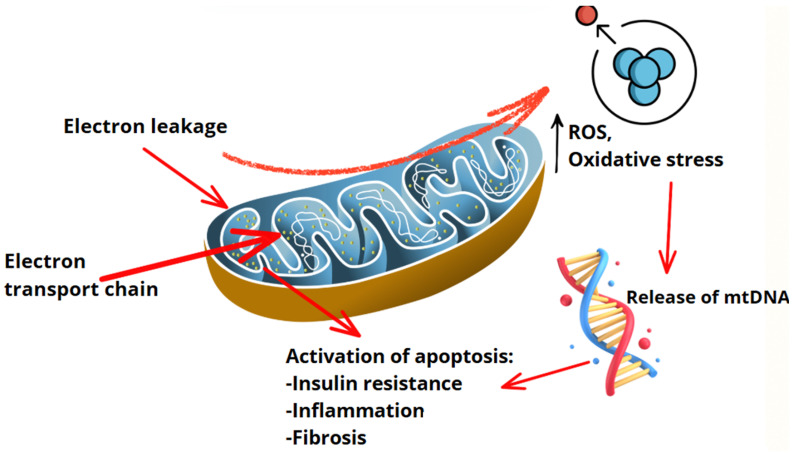
Overview of mitochondrial alterations observed in MetS. Electron leakage from the respiratory chain leads to excessive production of ROS, reduced ATP synthesis, and mtDNA release. These events contribute to redox imbalance, activation of apoptotic pathways, and stimulation of the NLRP3 inflammasome, triggering pro-inflammatory responses. Mitochondrial dysfunction is a central factor in insulin resistance, oxidative stress, and cellular damage in metabolic tissues, further contributing to the development of cardiovascular and metabolic diseases. ROS: Reactive Oxygen Species. Figure prepared with Canva.com.

As pointed out above, mitochondria are cellular organelles present in the cytoplasm of eukaryotic cells and are composed of two membranes, one internal and one external [124,125]. In their normal functioning, the primary function of these organelles is to generate energy, that is, to produce ATP through compounds such as sugars, amino acids, and fatty acids [126,127]. When these nutrients are transported into the mitochondrial membrane they undergo oxidation through the tricarboxylic acid cycle and beta oxidation [128]. During the degradation of these compounds, electrons are released and captured by the molecules adenine dinucleotide (NAD) and flavin adenine dinucleotide (FAD), which are then reduced to NADH and FADH_2_ [129,130]. After that, these molecules donate their electrons to the electron transport chain, which, in turn, generates ATP through ATP synthase after a series of events, thereby completing the process known as oxidative phosphorylation [131]. In MetS, the main mitochondrial alterations are ROS generation, membrane and mitochondrial DNA (mtDNA) damage, and decreased ATP production [132,133,134]. Beyond classical bioenergetic pathways, tryptophan–kynurenine metabolism may also connect inflammatory signaling to mitochondrial ATP maintenance, redox regulation, NAD^+^ metabolism, mitophagy, and inflammasome control [135,136]. Figure 2 shows an overview of mitochondrial alterations observed in MetS.

In individuals with this syndrome, adipocyte hypertrophy causes an overload during energy-generating processes in the organelle, especially during oxidative phosphorylation. This disruption of mitochondrial homeostasis results in electron leakage during NADH- and FADH_2_-mediated electron transfer, generating ROS. These reactive species remain unneutralized due to overloaded antioxidant defenses, ultimately leading to mitochondrial dysfunction [137,138]. In skeletal muscle, mitochondrial dysfunction induced by oxidative stress reduces lipid utilization as an energy source [139]. The normal and functional state of beta-oxidation is disturbed by ROS generation and, as a result, non-oxidized FFA, such as diacylglycerides and ceramides, accumulate in other tissues, modulating tissue cell signaling and generating insulin resistance [131,140,141,142]. In addition, AMP-activated kinase (AMPK) plays a crucial role in the generation of ATP within mitochondria [143,144]. This enzyme is activated when the available energy in the cell drops, that is, when ATP levels are low, and its primary function is to interrupt anabolic pathways and activate catabolic pathways [145,146]. In dysfunctional mitochondria, there is decreased AMPK synthesis, leading to increased lipogenesis and reduced FFA oxidation, factors that further increase insulin resistance in individuals with MetS [147,148,149].

A decrease in mtDNA is important for carrying out functions such as the production of constituents necessary for mitochondrial function, which is also associated with MetS [150,151]. Several copies of mtDNA are present within the mitochondrial matrix. Its alteration is susceptible in individuals with the syndrome because it is located near the electron transport chain and is susceptible to changes during oxidative stress [152,153,154,155]. The primary consequence of this alteration in mtDNA is a decrease in its copies, leading to deficiencies in OXPHOS proteins (oxidative phosphorylation) and activating the transcription factor-1 [107,156,157]. This factor is responsible for activating the polarization of M1 macrophages. Then, they will release a series of pro-inflammatory mediators, which, as a final result, lead to an increase in insulin resistance [158,159,160]. In addition to these changes, studies show that MetS is associated with decreased mitochondrial DNA copy number (mtDNA CN) and this change in mtDNA copy number is associated with an increased risk of type 2 diabetes [161,162]. Moreover, studies have proven a relationship between the formation of atherosclerotic plaques and a decrease in mtDNA [163,164,165].

During mitochondrial dysfunction, processes such as mitophagy (elimination of damaged mitochondria) are deregulated, leading to the activation of inflammasomes and further increasing the inflammatory state. As previously demonstrated, this scenario contributes to the formation of atherosclerotic plaques [166,167,168,169]. Furthermore, mtDNA is closely linked to cell death [170]. mtDNA is released into the cytosol under conditions of mitochondrial stress via signaling from the pro-inflammatory cytokines TNF-α and IL-β [154,171,172]. The mechanism by which this occurs is that, once mtDNA is recognized outside the mitochondrial matrix as a DAMP, it triggers a series of intracellular signaling processes that ultimately aim to induce apoptosis [173].

From a systemic perspective, renal cells with accelerated metabolism, which constantly require ATP, are also susceptible to mitochondrial dysfunction. Studies show that mitochondrial ROS production in tubular cells damages these cells, which are sensitive to toxicity, and alters mitochondrial energy production, resulting in cell death [174,175]. This factor, combined with the changes already mentioned in liver and heart tissues, suggests that MetS does not occur solely in adipose tissue, necessitating careful examination of the organs affected [176,177,178].

Beyond their endocrine actions on distant organs, organokines have emerged as critical regulators of mitochondrial homeostasis, directly influencing mitochondrial biogenesis, dynamics, mitophagy, oxidative phosphorylation, and cellular metabolic adaptation. Under physiological conditions, several protective organokines contribute to the maintenance of mitochondrial fitness by activating signaling pathways involved in energy sensing and mitochondrial quality control. Conversely, chronic metabolic stress disrupts the balance between protective and pathological organokines, promoting mitochondrial dysfunction that further amplifies metabolic inflammation and cardiometabolic disease progression [179,180].

One of the major mechanisms involves the regulation of mitochondrial biogenesis through the AMP-activated protein kinase (AMPK)–Sirtuin 1 (SIRT1)–peroxisome proliferator-activated receptor gamma coactivator-1 alpha (PGC-1α) signaling axis. Protective organokines such as adiponectin, irisin, fibroblast growth factor 21 (FGF21), and osteocalcin stimulate AMPK activation, leading to increased PGC-1α activity and subsequent induction of nuclear respiratory factors (NRF1 and TFAN) and mitochondrial transcription factor A (TFAM). These transcriptional regulators coordinate mitochondrial DNA replication, mitochondrial protein synthesis, oxidative phosphorylation, and ATP production, thereby preserving cellular bioenergetic capacity and metabolic flexibility. In contrast, reduced circulating levels of these organokines during MetS impair mitochondrial biogenesis, contributing to defective energy metabolism in adipose tissue, skeletal muscle, liver, and the cardiovascular system [181,182,183,184].

Organokines also influence mitochondrial quality control by regulating mitochondrial dynamics and mitophagy. Mitochondrial integrity depends on a dynamic balance between fusion and fission processes mediated by mitofusins (MFN1 and MFN2), optical atrophy protein 1 (OPA1), and dynamin-related protein 1 (DRP1). Protective organokines promote balanced mitochondrial remodeling and facilitate the selective removal of dysfunctional mitochondria through PINK1/Parkin-mediated mitophagy, preventing the accumulation of damaged organelles and limiting excessive reactive oxygen species (ROS) production. However, chronic exposure to pathological organokines, including resistin, leptin resistance-associated signaling, fetuin-A, c, and certain pro-inflammatory hepatokines, disrupts mitochondrial dynamics, suppresses mitophagy, and favors the accumulation of dysfunctional mitochondria characterized by reduced oxidative phosphorylation efficiency and increased electron leakage [185,186,187].

The resulting mitochondrial dysfunction promotes excessive mitochondrial ROS generation, lipid peroxidation, oxidative damage to mtDNA, and activation of redox-sensitive inflammatory pathways such as NF-κB and the NLRP3 inflammasome. This creates a vicious cycle in which mitochondrial oxidative stress further stimulates the production of pro-inflammatory organokines and cytokines, amplifying systemic metabolic inflammation, insulin resistance, endothelial dysfunction, and cardiovascular remodeling. Furthermore, impaired mitochondrial ATP production compromises insulin signaling, substrate utilization, and metabolic flexibility, limiting the ability of metabolically active tissues to adapt to fluctuations in nutrient availability and energetic demand [188,189].

Importantly, mitochondrial dysfunction is not merely a downstream consequence of altered organokine secretion but also actively contributes to endocrine dysregulation. Mitochondrial stress modifies adipokine, myokine, hepatokine, and cardiokine secretion profiles, reinforcing maladaptive interorgan communication and establishing a feed-forward loop that perpetuates chronic inflammation and progressive cardiometabolic deterioration. Therefore, preservation of mitochondrial fitness represents a central mechanism through which organokines coordinate whole-body metabolic homeostasis and may constitute an attractive therapeutic target for interrupting the reciprocal interactions linking MetS to cardiovascular disease [190,191].

In summary, mitochondrial dysfunction further amplifies the detrimental effects of organokine dysregulation by increasing mitochondrial ROS production, impairing endothelial energy metabolism, and activating redox-sensitive inflammatory pathways, including NF-κB and the NLRP3 inflammasome. Persistent oxidative stress disrupts vascular homeostasis, promotes endothelial senescence, and contributes to arterial stiffness and hypertension. Therefore, mitochondrial dysfunction and altered organokine signaling act synergistically to accelerate cardiovascular injury in MetS.

All together, these findings indicate that organokine imbalance should not be viewed solely as a consequence of MetS but rather as a major upstream regulator of mitochondrial homeostasis. Through coordinated effects on mitochondrial biogenesis, mitophagy, redox balance, and metabolic adaptation, organokines integrate nutrient sensing with interorgan communication, ultimately determining cardiometabolic resilience or disease progression. Thus, restoring the protective organokine network may offer a dual therapeutic opportunity, simultaneously preserving mitochondrial fitness and breaking the vicious cycle of meta-inflammation that fuels cardiometabolic disorders.

## 5. Gut Microbiota-Derived Metabolites as Modulators of Metainflammation and Mitochondrial Dysfunction

In general, humans and the intestinal microbiota form a symbiotic relationship of commensalism, orchestrated by positive or negative feedback mechanisms [192,193,194]. The microbiome is an intestinal system of microbial mass, which means it is composed of bacteria and their metabolic activities [195,196,197]. The importance of this system lies mainly in its ability to digest non-digestible substrates, such as dietary fiber [198]. Through the fermentation of these carbohydrates, these microorganisms produce short-chain fatty acids (SCFAs). Among these are butyrate, acetate, and propionate, which are part of a group that has 1 to 6 saturated carbons in its composition [199,200]. Through mechanisms that regulate energy expenditure, these SCFAs balance glucose metabolism, influence appetite, modulate the immune system, and directly impact metabolic health [201,202]. Among their primary functions, these SCFAs provide energy to colonocytes, ensuring the system’s sustainability. Additionally, they can be absorbed and enter the peripheral circulation via the portal system, thereby ensuring systemic effects [203]. The high production of these metabolites may be related to the prevention of gastrointestinal dysfunctions and other metabolic disorders, such as obesity. Conversely, the opposite is also observed [204]. In the gastrointestinal tract, SCFAs function as signaling molecules by activating G protein-coupled receptors (GPCRs), mainly FFAR2 (GPR43) and FFAR3 (GPR41), expressed on enteroendocrine L cells. This signaling promotes the release of glucagon-like peptide-1 (GLP-1) and peptide YY (PYY), hormones that regulate satiety, intestinal motility, insulin secretion, and systemic energy homeostasis [205,206]. Furthermore, when activated, these receptors promote the activation of immune system cells, leading to the release of prostaglandin E2 and IL-10 which together mediate an anti-inflammatory action and regulate intestinal cell proliferation [207].

**Figure 3:**
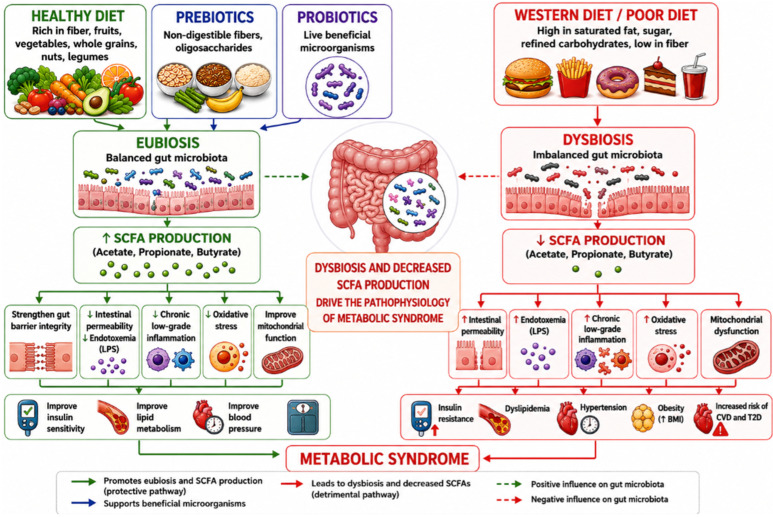
Gut microbiota, SCFAs, and their impact on metabolic health. The role of the gut microbiota in regulating metabolic health through the production of SCFAs (acetate, propionate, and butyrate). SCFAs improve insulin sensitivity, lipids, and blood pressure; reduce BMI, modulate appetite, and reduce systemic inflammation, oxidative stress, and mitochondrial dysfunction. Dysbiosis and decreased SCFA production contribute to the pathophysiology of MetS. BMI: body mass index; SCFAs: short-chain fatty acids. Figure prepared with Canva.com and improved by GPT.com.

In the case of MetS, several authors have reported an association between decreased SCFA-producing bacteria and increased pathological mechanisms of the disease [208,209]. By binding to GPCRs (e.g., GPR43), these metabolites activate pathways that stimulate GLP-1 secretion. As a multifunctional incretin hormone, GLP-1 enhances insulin sensitivity and promotes its secretion from pancreatic β-cells, directly countering insulin resistance, a key determinant of MetS [210,211,212,213]. In addition, its action on pancreatic beta cells promotes insulin secretion, which also helps regulate serum glucose levels [214]. Evidence for this lies in the high incidence of MetS risk factors in Western populations where dietary patterns are characterized by low fiber intake [215]. Critically, GPR43 activation inhibits insulin-mediated lipid and glucose deposition in adipose tissue. Consequently, a deficiency in SCFAs, which stimulates GPR43, promotes ectopic fat accumulation while reducing muscle utilization of these substrates. This process directly contributes to abdominal obesity, a core determinant of MetS [15,216,217]. Thus, it is evident that the intestinal microbiota and its mechanisms of self-regulation and metabolic regulation influence systemic factors that determine predisposition, the presence of risk factors, and even the severity of MetS. This is why the microbiome is also strongly associated with possible treatments for MetS, seeking to maintain this system as a path to metabolic balance [218,219,220,221]. Figure 3 summarizes the role of gut microbiota, SCFAs, and MetS.

Several studies have found that supplementation with probiotics will increase the amount of intestinal bacteria that produce SCFAs and it is a solution for reducing risk factors for the syndrome. The administration of *Bifidobacterium lactis* was associated with a reduction in hepatic gluconeogenesis and improved translocation of the glucose transporter-4 (GLUT4), reducing insulin resistance [207,222,223,224]. In addition, the increase in fiber intake creates an environment conducive to the proliferation of these bacteria, thereby positively influencing the stabilization of the fermentation process and the production of SCFAs. Thus, increased fiber intake is positively associated with reduced body adiposity which directly influences MetS [225,226]. For these reasons, studies indicate that factors associated with greater GLP-1 release, such as the high production of SCFAs by the intestinal microbiota through fiber intake, are directly related to MetS [227]. Furthermore, studies indicate that factors associated with greater GLP-1 release, such as the high production of SCFAs by the intestinal microbiota through fiber intake, are directly associated with attenuation of MetS and a reduction in its risk factors for future cardiovascular and metabolic diseases. Collectively, these findings suggest that dietary fiber, SCFA-producing bacteria, and GLP-1-mediated signaling form a microbiota–metabolic axis that may attenuate insulin resistance, adiposity, and cardiovascular risk in MetS [75,228,229,230].

## 6. Organokine-Mediated Interorgan Crosstalk in MetS

**Figure 4:**
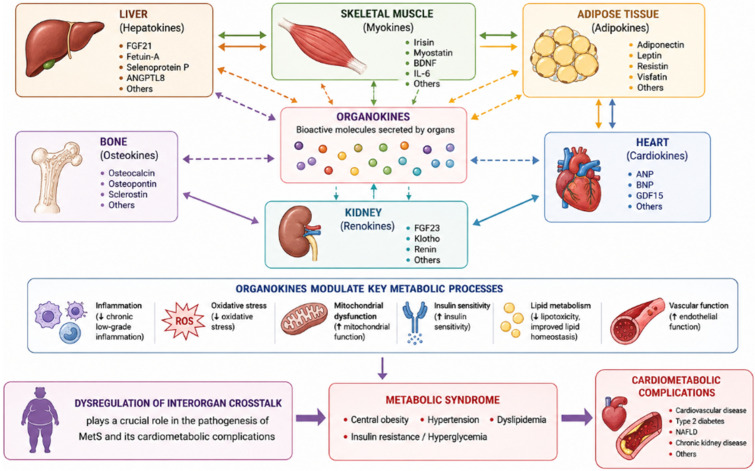
Interorgan crosstalk in metabolic syndrome (MetS). Interorgan communication mediated by organokines, bioactive molecules secreted by metabolically active organs, including skeletal muscle (myokines), liver (hepatokines), adipose tissue (adipokines), heart (cardiokines), bone (osteokines), and kidney (renokines). Through endocrine, paracrine, and autocrine signaling, these organokines regulate inflammation, oxidative stress, mitochondrial function, insulin sensitivity, lipid metabolism, and vascular homeostasis. In MetS, chronic low-grade inflammation and metabolic stress disrupt organokine secretion, leading to impaired interorgan communication that contributes to insulin resistance, dyslipidemia, endothelial dysfunction, and the progression of cardiometabolic complications. Abbreviations: MetS, metabolic syndrome; FGF21, fibroblast growth factor 21; ANGPTL8, angiopoietin-like protein 8; BDNF, brain-derived neurotrophic factor; IL-6, interleukin-6; ANP, atrial natriuretic peptide; BNP, B-type natriuretic peptide; GDF15, growth differentiation factor 15; FGF23, fibroblast growth factor 23; ROS, reactive oxygen species; NAFLD, non-alcoholic fatty liver disease. Figure prepared with Canva.com and improved by GPT.com.

In individuals with MetS, chronic low-grade inflammation, driven by sustained positive energy balance (high caloric intake and reduced expenditure), alters the secretion profiles of hepatokines, myokines, adipokines, and cardiokines [231]. Dysregulation of the release of these proteins strongly influences the development and progression of MetS and, consequently, cardiovascular diseases [231,232,233,234,235] (Figure 4).

### 6.1. Adipokines: Adipose Tissue Inflammation and Insulin Resistance

Since its discovery, studies on adiponectin, leptin, adipsin, and other adipose tissue secretions have advanced, particularly regarding the organ’s importance in regulating metabolism. Adipokines are protein hormones released by adipocytes in response to the body’s metabolic conditions. In general, they perform their functions by regulating glucose and lipid metabolism, modulating immune responses, and regulating the body’s response to insulin [236,237,238]. Due to their fundamental role in metabolic homeostasis, several pharmacological and therapeutic interventions targeting adipose tissue are being developed for the treatment of obesity, type 2 diabetes, cardiovascular disease, and MetS.

Leptin is a crucial ally in weight loss, as it suppresses hunger, increases energy expenditure, and influences the immune system. In obese individuals, as noted above, the body may exhibit leptin resistance [239,240]. High circulating levels of resistin are associated with metabolic disorders, indicating the presence of inflammation and insulin resistance [241,242]. Adiponectin has beneficial effects on the body by promoting insulin sensitivity, inhibiting the formation of atherosclerotic plaques, and exerting anti-inflammatory effects. However, its levels are low in people with obesity and MetS [243,244].

### 6.2. Hepatokines: Liver-Derived Regulators of Lipid and Glucose Homeostasis

In liver tissue, hepatokines influence metabolism and are associated with the pathogenesis of MetS. In general, these molecules are capable of controlling lipid and glucose metabolism, maintaining whole-body homeostasis, increasing insulin resistance, and acting as pro-inflammatory factors [245,246]. These functions highlight the liver’s crucial role in the development of metabolic disorders associated with overnutrition. In MetS, hepatokines are linked to increased oxidative stress, endoplasmic reticulum stress, altered insulin signaling, altered lipid profiles, mitochondrial dysfunction, and lipid accumulation in the liver and skeletal muscle [247,248,249,250].

In addition to being secreted by various tissues such as the liver, pancreas, muscle, and adipose tissue, fibroblast growth factor-21 (FGF-21) plays essential roles in metabolism. FGF-21 increases glucose uptake by adipose tissue, exhibits anti-inflammatory effects, and also participates in adipose tissue browning [251,252]. Growth differentiation factor 15 (GDF-15) is associated with appetite regulation and increased insulin sensitivity, and contributes to weight loss by increasing energy expenditure [253,254]. Fetuin-A is widely used as a marker for metabolic diseases, especially type 2 diabetes mellitus (T2DM), as its action is associated with increased insulin resistance [255,256]. Angiopoietin-like 4 (ANGPTL4) has been associated with stimulating lipolysis and is also responsible for fat redistribution [218,257].

### 6.3. Myokines: Skeletal Muscle Metabolism and Exercise-Responsive Signaling

Skeletal muscle has essential functions in the body; in addition to producing mechanical energy and influencing basal metabolism, it also exhibits secretory activity, releasing myokines [219,220,221]. These molecules are bioactive peptides released by muscles and exert their functions through autocrine, paracrine, and endocrine effects. Its main functions include increasing insulin sensitivity, regulating lipolysis, inflammation, and metabolic homeostasis. These factors highlight physical exercise and increased muscle mass as important allies in the treatment and prevention of MetS [222,223,224]. For example, irisin is responsible for the browning of brown adipose tissue and also exhibits anti-inflammatory properties [225,226]. IL-6 released after physical exercise has been shown to affect lipid metabolism by increasing lipolysis, and it is also associated with increased glucose uptake in muscle. Myonectin has effects on the oxidation of FFA and also increases glucose uptake by skeletal muscle [227,228].

### 6.4. Cardiokines: Cardiac Stress Signals and Cardiometabolic Remodeling

Similar to liver and adipose tissue, skeletal muscle and cardiac tissue also have endocrine actions. Cardiokines, proteins secreted by cardiomyocytes, have beneficial effects on regulating metabolism, such as stimulating lipolysis, and play a crucial role in systemic anti-inflammatory responses. Cardiokines influence cardiac stress, reduced muscle mass, atherosclerosis, diabetic cardiomyopathy, and heart failure [258,259,260,261]. For example, atrial natriuretic peptide (ANP) and B-type natriuretic peptide (BNP) are the main cardiokines released by the heart. These hormones are cardioprotective and have recently been identified at reduced levels in obese individuals. They affect lipid and FFA metabolism and are linked to improved insulin sensitivity [262,263].

Myostatin, found in high levels in individuals with MetS, has the ability to alter mitochondrial function, thereby affecting lipid metabolism, increasing insulin resistance, and decreasing muscle mass [264]. Myonectin is responsible for increasing the uptake of FFA by adipose and hepatic tissue and may be associated with insulin resistance when circulating at low levels [265,266]. Therefore, new treatments targeting the adipomyocardiokine axis may help treat cardiovascular disease, obesity, and MetS (MetS), as these tissues communicate and influence one another.

### 6.5. Osteokines and Renokines: Bone–Kidney Contributions to Systemic Metabolic Dysfunction

In the bones, osteokines exert a significant influence on MetS through multiple mechanisms [267]. Their actions involve regulating insulin sensitivity and glycemic homeostasis. Examples include osteocalcin, which stimulates pancreatic beta-cell proliferation and insulin secretion [268]. Furthermore, it promotes increased glucose uptake in muscle and adipose tissue via activation of the AMPK/FOXO1 pathway. It reduces pro-inflammatory cytokines, such as TNF-α and IL-6, and oxidative stress. It reduces lipid accumulation and activates antioxidant pathways, such as the Nrf2 pathway. It also aggravates hepatic and systemic inflammation [269,270].

Sclerostin inhibits the Wnt/β-catenin pathway, impairing insulin signaling and contributing to insulin resistance [271]. Fibroblast Growth Factor 23 (FGF-23), when elevated, is associated with hepatic steatosis; it induces vascular calcification and endothelial dysfunction, increasing the risk of hypertension and cardiovascular events [15]. Neutrophil gelatinase-associated lipocalin (NGAL), also known as lipocalin-2, improves glucose intolerance via PPARγ, and in excess, it exacerbates inflammation. It also modulates hepatic adipogenesis, influencing abdominal circumference [272]. Therefore, an imbalance in osteokines promotes insulin resistance, dyslipidemia, chronic inflammation, and hepatic steatosis, amplifying the components of MetS.

As previously mentioned, individuals with MetS experience systemic changes, including kidney involvement. When released, renokines primarily affect glucose and lipid metabolism, but they also influence blood pressure through the renin–angiotensin–aldosterone system (RAAS) [273,274]. For example, erythropoietin, in addition to its role in erythrocyte production, has been associated with improved insulin sensitivity and vascular endothelial protection and may even aid in weight loss [275,276]. Renin, through the RAAS, has been strongly associated with hypertension in patients with MetS; it also has effects on oxidative stress and insulin resistance [277,278]. NGAL is known to be a marker of cell injury, and elevated levels of this biomarker are present in people with diabetic nephropathy [279]. Furthermore, NGAL participates in immune responses, and although its involvement in glucose homeostasis and lipogenesis in individuals with MetS is not yet fully understood, this molecule may play a role in these processes [280,281].

In summary, these biomarkers are essential for the establishment and progression of MetS, since, in general, when released, myokines, hepatokines, adipokines, cardiokines, osteokines and renokines are responsible for increased insulin resistance, modulation of adipose tissue with increased lipolysis, changes in glucose metabolism, increased pro-inflammatory cytokines, hypertension, and heart, liver or kidney disease.

Different organokines play a specific role in MetS. Table 1 summarizes their general functions and the effects related to MetS.

### 6.6. Integrated Organokine Crosstalk in MetS

In a scenario of systemic inflammation and insulin resistance, all organokines (hepatokines, myokines, cardiokines, adipokines, osteokines, and rinokines) are involved in the regulation of inflammation, lipid and glucose metabolism, and insulin sensitivity. MetS is related to an imbalance in these molecules that promote insulin resistance (thanks to the inhibition of signaling pathways, such as Wnt/β-catenin and AMPK), mitochondrial dysfunction, and oxidative stress; accumulation of lipids in tissues such as liver, muscle, and heart; and hypertension and cardiovascular diseases [337,338].

In the muscle–liver–adipose tissue axis, it is possible to mention that myokines (such as irisin and myostatin) and hepatokines (such as FGF-21) can modulate the browning of adipose tissue, increasing energy expenditure and reducing adiposity. Adipokines (such as leptin and adiponectin) regulate fatty acid oxidation in muscle and liver. FGF-21 (secreted by liver, muscle, and adipose tissue) acts as a central metabolic regulator, improving insulin sensitivity and lipid homeostasis [24,25].

**Figure 5:**
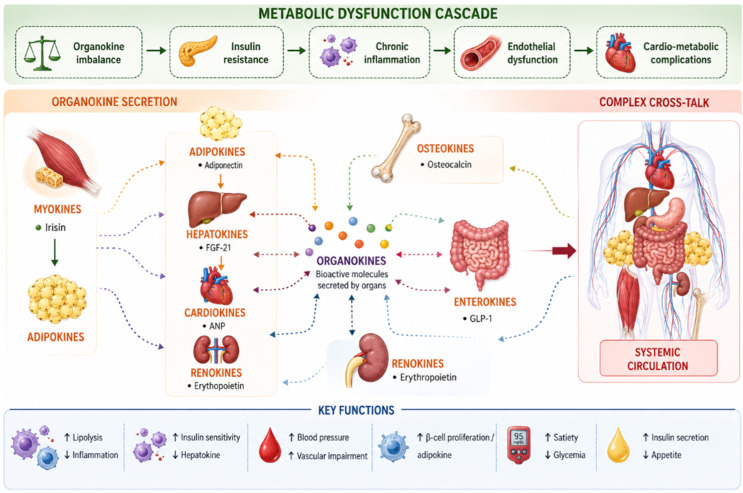
Illustration of interorgan communication mediated by organokines, bioactive molecules secreted by various organs involved in metabolic regulation. Skeletal muscle (myokines), liver (hepatokines), adipose tissue (adipokines), heart (cardiokines), bone (osteokines), kidney (renokines), and intestine (enterokines) release signaling molecules such as irisin, FGF-21, adiponectin, ANP, osteocalcin, erythropoietin, and GLP-1. These organokines modulate inflammation, insulin sensitivity, lipid metabolism, and vascular function. Dysregulation of this interaction plays a crucial role in the pathogenesis of MetS and its cardiometabolic complications. Abbreviations: ANP: atrial natriuretic peptide; GLP-1: Glucagon-like peptide 1; FGF-21: fibroblast growth factor 21. Figure prepared with Canva.com and improved by GPT.com.

As seen above, some molecules can be secreted by multiple tissues and have integrated roles that are summarized in Table 2 and Figure 4 and Figure 5.

### 6.7. Organokine Network Imbalance: Beyond Isolated Alterations in Circulating Levels

An emerging concept in MetS is that disease progression is not determined solely by changes in the circulating concentrations of individual organokines but rather by a profound disruption of the physiological balance among protective and pathological endocrine mediators. Under healthy conditions, organokines released by adipose tissue, skeletal muscle, liver, bone, heart, kidney, and the nervous system act in a coordinated manner to maintain metabolic homeostasis through the regulation of energy expenditure, insulin sensitivity, mitochondrial fitness, substrate utilization, redox balance, and inflammatory responses. However, chronic nutrient excess, obesity, physical inactivity, and aging progressively disrupt this finely regulated endocrine network, leading to an imbalance characterized by reduced secretion or impaired responsiveness to metabolically beneficial organokines, together with persistent elevation of pro-inflammatory and metabolically detrimental mediators. This endocrine disequilibrium compromises interorgan communication, exacerbates mitochondrial dysfunction, promotes oxidative stress and chronic low-grade inflammation, and accelerates the development of insulin resistance, endothelial dysfunction, and cardiovascular complications. Therefore, MetS should be viewed as a disorder of organokine network dysregulation, in which the loss of coordinated endocrine signaling is more relevant than isolated alterations in individual organokines [191,339,340,341] (Figure 4 and Figure 5).

#### 6.7.1. Protective Versus Pathological Organokines

Although many organokines exhibit context-dependent biological activities, they can be broadly categorized according to their predominant effects on metabolic homeostasis. Protective organokines—including adiponectin, irisin, FGF21, ANP, osteocalcin, and several cardiokines and batokines—generally enhance insulin sensitivity, stimulate AMPK/SIRT1/PGC-1α signaling, promote mitochondrial biogenesis and oxidative metabolism, improve endothelial function, and suppress chronic inflammation. In contrast, persistent elevation of pathological organokines, such as resistin, fetuin-A, myostatin, NGAL, and conditions characterized by leptin resistance, contributes to insulin resistance, mitochondrial dysfunction, oxidative stress, activation of NF-κB and NLRP3 inflammasome pathways, vascular inflammation, and adverse cardiovascular remodeling. Importantly, several organokines may exert either protective or detrimental effects depending on tissue source, circulating concentration, disease stage, receptor sensitivity, and metabolic context. Thus, the pathological hallmark of MetS is not the action of a single organokine but the disruption of the dynamic equilibrium within the organokine network [342,343].

These observations support the concept that MetS represents a state of organokine network imbalance, in which the progressive loss of beneficial endocrine signaling and the predominance of pathological mediators reinforce metainflammation, impair mitochondrial fitness, and establish a vicious cycle that drives cardiometabolic disease progression.

#### 6.7.2. Cardiovascular Consequences of Organokine Imbalance

To summarize, we can say that the cardiovascular consequences of organokine imbalance extend beyond endothelial dysfunction and involve multiple pathological processes. Dysregulated adipokines, including reduced adiponectin and increased leptin and resistin, favor vascular inflammation, hypertension, and atherosclerosis. Elevated hepatokines such as fetuin-A promote insulin resistance, vascular calcification, and endothelial dysfunction, whereas altered cardiokines may impair myocardial metabolism and contractile function. Likewise, dysregulated osteokines and renokines contribute to vascular calcification, myocardial remodeling, fibrosis, and heart failure. Collectively, these alterations establish a chronic pro-inflammatory and pro-oxidative environment that accelerates cardiovascular disease progression in individuals with MetS.

Importantly, these cardiovascular abnormalities should not be interpreted as the consequence of isolated changes in individual organokines but rather as the result of a complex disruption of interorgan communication. The convergence of mitochondrial dysfunction, oxidative stress, chronic low-grade inflammation, and organokine imbalance establishes a self-perpetuating cycle that promotes endothelial dysfunction, vascular remodeling, myocardial injury, and progression of cardiovascular disease. This integrative perspective reinforces the concept that restoring physiological organokine signaling may represent a promising strategy to reduce cardiovascular risk in MetS.

## 7. Integrated Model Linking MetS to Cardiovascular Disease

The adipo-myo-cardiac axis may include adipokines (leptin and resistin) and myokines (IL-6 and irisin) that regulate fatty acid oxidation and insulin sensitivity in muscle and the heart. Cardiokines (ANP and BNP) modulate the release of adiponectin by adipose tissue [24,25,71,72,83] (Figure 4).

The bone-kidney-liver axis includes osteokines (osteocalcin and FGF-23) and rinokines (erythropoietin and Klotho) that regulate mineral metabolism, inflammation, and vascular function. FGF-23 (osteokine) and renin are linked to vascular calcification and hypertension in MetS [55,58,112].

The metabolic pattern in patients with MetS leads to systemic dysfunction of organokines. Hepatokines (such as Fetuin-A) and adipokines (such as leptin) exacerbate insulin resistance and hepatic steatosis [19,28,36]. Cardiokines and myokines (such as IL-6) contribute to cardiac dysfunction and muscle loss [41,79]. Osteokines (such as sclerostin) and rinokines (such as renin) promote hypertension and kidney disease [55,61].

As observed above, MetS is a result of a complex cross-talk among hepatokines, myokines, cardiokines, adipokines, rinokines, and osteokines. Dysfunctions in these axes promote an oxidative environment, chronic inflammation, insulin resistance, metabolic imbalance, and cardiometabolic complications.

The evidence summarized in this review supports an integrated model in which nutrient excess and adipose tissue expansion initiate metainflammation, leading to oxidative stress, mitochondrial dysfunction, and altered secretion of organokines. Mitochondrial impairment amplifies ROS production, ATP deficiency, mtDNA release, inflammasome activation, and endothelial dysfunction, while dysregulated adipokines, myokines, hepatokines, cardiokines, osteokines, and renokines propagate metabolic stress across organs. Gut microbiota dysbiosis and reduced SCFA production further weaken immunometabolic homeostasis. Together, these processes create a self-reinforcing network that links MetS to atherosclerosis, hypertension, insulin resistance, renal injury, hepatic steatosis, and cardiovascular disease progression.

## 8. Conclusions

MetS should be understood not merely as a cluster of cardiometabolic risk factors, but as a self-perpetuating network of metainflammation, mitochondrial dysfunction, oxidative stress, organokine imbalance, and microbial dysregulation. Metainflammation contributes to insulin resistance, vascular damage, and energy imbalance, ultimately promoting the onset and progression of cardiovascular disease. Mitochondrial dysfunction, a central feature of MetS, exacerbates ROS production, impairs ATP synthesis, and activates apoptotic and inflammatory signaling pathways, further exacerbating metabolic and cardiovascular imbalance. In this context, organokines, bioactive molecules secreted by the liver, muscle, adipose tissue, heart, bone, kidneys, and intestine, emerge as key inter-organ modulators for homeostasis or disease progression. Their dysregulated secretion promotes a pro-inflammatory, atherogenic, and insulin-resistant state. Furthermore, alterations in the gut microbiota, especially the reduction of short-chain fatty acids, disrupt immunometabolic homeostasis and contribute to the metabolic dysregulation observed in MetS. This integrated perspective shifts the focus from isolated metabolic abnormalities to interconnected signaling networks, supporting the development of biomarker-guided, multi-target, and organ-specific strategies for preventing cardiovascular complications in MetS.

## 9. Clinical and Translational Perspectives

The growing understanding of organokine-mediated interorgan communication has opened new opportunities for the prevention and treatment of MetS. Rather than considering insulin resistance, chronic inflammation, mitochondrial dysfunction, and dyslipidemia as isolated pathological processes, current evidence supports the concept that these alterations are interconnected through a complex network of endocrine signaling among metabolic organs. Consequently, therapeutic strategies capable of restoring organokine balance may provide broader cardiometabolic benefits than interventions targeting individual metabolic abnormalities [9,24,32].

Lifestyle modification remains the cornerstone of MetS management. Many studies have demonstrated that structured exercise programs and dietary patterns improve insulin sensitivity, reduce chronic low-grade inflammation, enhance endothelial function, and promote favorable changes in adipokine and myokine secretion. Exercise interventions have also been associated with increased circulating irisin and adiponectin levels, reduced leptin concentrations, improved mitochondrial function, and attenuation of oxidative stress, reinforcing the importance of organokine-mediated adaptations [7,221,324,325].

Pharmacological therapies have further expanded the therapeutic landscape. GLP-1 receptor agonists have demonstrated substantial reductions in body weight and improvements in glycemic control and cardiovascular risk factors. More recently, dual GIP/GLP-1 receptor agonists such as tirzepatide produced even greater improvements in weight reduction and insulin sensitivity. In addition, SGLT2 inhibitors have consistently reduced cardiovascular and renal events in high-risk patients while exerting favorable effects on inflammation, oxidative stress, and mitochondrial function, mechanisms that may partly involve modulation of organokine signaling [16,196,197,198,199,343,344].

Emerging evidence also suggests that therapeutic approaches targeting gut microbiota composition, SCFA production, mitochondrial function, and chronic metainflammation may complement conventional metabolic therapies [14,24]. Furthermore, advances in multi-omics technologies are facilitating the identification of circulating organokines as potential biomarkers for disease progression, cardiovascular risk stratification, and treatment response. Integrating clinical phenotyping with metabolomics, proteomics, transcriptomics, and organokine profiling may enable more precise therapeutic decisions and accelerate the implementation of personalized medicine in patients with MetS. Although further longitudinal and interventional studies are required, targeting organokine-mediated interorgan communication represents a promising translational strategy for reducing the burden of cardiometabolic disease.

## 10. Future Perspectives

Future research should move beyond single-pathway models of MetS and adopt multidimensional strategies that capture the interaction among metainflammation, mitochondrial dysfunction, organokine signaling, gut microbiota, and cardiovascular injury. First, organokines should be systematically evaluated as early biomarkers, diagnostic tools, and therapeutic targets, particularly in individuals transitioning from metabolically unhealthy obesity to overt cardiovascular disease. Second, mitochondrial-targeted interventions that restore redox balance, improve ATP synthesis, preserve mtDNA integrity, and limit inflammasome activation deserve further translational investigation. Third, modulation of gut microbiota composition and SCFA production may provide an additional strategy to restore immunometabolic homeostasis.

Systems biology, multi-omics profiling, and network-based approaches will be essential for mapping the crosstalk among adipose tissue, skeletal muscle, liver, heart, bone, kidney, and intestine. Clinical trials should test combined interventions, including exercise, dietary modulation, microbiota-directed therapies, and agents targeting FGF-21, myostatin, adiponectin, or related organokine pathways. Ultimately, personalized cardiometabolic medicine may help identify patient subgroups that benefit from systemic versus organ-specific interventions, improving therapeutic efficacy while minimizing adverse effects.

## Figures and Tables

**Table 1:** Main characteristics of organokines and their role in the pathophysiology of MetS.

Organokine	Molecule	General Functions	Role in Metabolic Syndrome	References
Myokines	Myonectin	-Neutralizes insulin resistance; -Regulates protein synthesis; -Cardioprotective factor; -Regulation of FFA metabolism; -Acts on glucose metabolism.	-Improved fat mobilization through physical exercise; -Increased levels may indicate the presence of type 2 diabetes and IR.	[282,283,284,285]
	FGF-21	-Regulates metabolic stress; -Activates autophagic pathways.	-Acts on glucose and lipid metabolism.	[286,287]
	Myostatin	-Acts on skeletal muscle fiber, limiting its growth; -Acts on bone.	-Increases insulin resistance; -Target-therapeutic for the treatment of Mets through its inhibition.	[288,289,290]
	Irisin	-Related to mitochondrial biogenesis; -Conversion of white adipose tissue to brown adipose tissue; -Increases thermogenesis.	-Acts on oxidative stress; -Reduces inflammation; -Improvement in lipid profile; -Increases insulin sensitivity.	[291,292]
	IL-6	-Acts on glucose homeostasis; -Promotes muscle hypertrophy.	-Increases lipolysis; -Increases oxidation of FFA; -Increases ROS production in adipose tissue; -Associated with mitochondrial dysfunction.	[216,293]
Hepatokines	Fetuin-A	-Increases lipolysis mainly in adipocytes; -Stimulates the inflammatory state in macrophages and adipocytes; -Increases insulin resistance.	-Increased levels in people with the prognosis; -Marker linking obesity and MetS; -Promotes the release of pro-inflammatory cells; -Increases the risk of developing type 2 diabetes and hypertension.	[294,295,296]
	FGF-21	-Stimulates lipolysis; -Used for the diagnosis and treatment of obesity and type 2 diabetes; -Promotes the oxidation of FFA in the liver.	-Improves insulin sensitivity; -Improves in lipid profile; -Promotes browning of adipose tissue; -Regulates metabolic homeostasis in cells.	[297,298]
	Activin E	-Increases thermogenesis; -Reduces lipolysis.	-Increases insulin sensitivity; -Changes the distribution of fat in the body.	[299]
	ANGPTL4	-Important role in lipid mobilization and metabolism; -Inhibits pancreatic lipases; -Stimulates lipolysis.	-Increases insulin resistance; -Increased levels in people with a disease prognosis; -Reduces fat absorption.	[300,301]
	Selenoprotein	-Promotes liver inflammation.	-Increases insulin resistance.	[302]
Adipokines	Adiponectin	-Increases oxidation of FFAs; -Increases lipolysis; -Acts in the protection of blood vessels.	-Reduces inflammation; -Increases insulin sensitivity; -Antiatherogenic effect; -Reduced levels in obese people.	[11,302]
	Resistin	-Used as a biomarker for atherosclerosis, cancer, inflammation, and other cardiovascular diseases; -Reduces the body’s energy expenditure.	-Increases insulin resistance; -Promotes inflammation in adipose tissue; -Increased levels in people with obesity.	[303,304]
	Visfatin	-Promotes lipolysis; -Promotes the increase in fatty acids in the liver.	-Increases insulin resistance; -Alters oxidative stress; -Promotes inflammation.	[305]
	Lepitin	-Increases oxidation of FFAs; -Suppresses appetite; -Increases thermogenesis.	-Increases inflammation; -May be at increased levels in people with Mets, indicating leptin resistance; -Increases systemic energy expenditure.	[306,307]
	Omentin	-Increases insulin sensitization; -Anti-inflammatory action; -It acts by reducing oxidative stress; -It has anti-atherosclerotic effects and cardioprotective effects; -Decreases leukocyte adhesion to the endothelium.	-Low levels in people with MetS; -Low levels in people with the syndrome indicate increased inflammation; -Low level of NO availability; -Increases oxidative stress; -Increases endothelial dysfunction; -Increase apoptosis.	[308,309,310,311,312]
Cardiokines	Natriuretic Peptides	-Induces lipid metabolism; -Increases lipid mobilization in adipose tissue; -Increases the metabolism of brown adipose tissue; -Improvement in the browning process of adipose tissue.	-Low circulating levels; -Low levels may be a risk for type 2 diabetes; -Improves insulin sensitivity; -Increases peptide secretion is related to positive feedback on adiponectin secretion.	[313,314,315]
	Myostatin	-Limits muscle growth; -Promotes the accumulation of lipids in the liver.	-Inhibits glucose uptake; -Reduces energy expenditure.	[316,317]
	BNP	-Promotes browning of WAT; -Regulates lipid metabolism; -Cardioprotective effect.	-Increases insulin sensitivity; -Promotes increased energy expenditure.	[318,319]
	ANP	-Cardioprotective effect; -Promotes browning of WAT; -Regulates lipid metabolism; -Participates in the autophagy of cardiac cells.	-Increases insulin sensitivity; -Promotes increased energy expenditure; -Regulation of lipid metabolism; -Regulates the release of adipokines.	[320]
	MED-13	-Increases oxygen consumption; -Promotes the oxidation of fatty acids.	-Increases insulin sensitivity; -Promotes mass gain; -Regulation of lipid metabolism; -Participates in adipocyte hypertrophy.	[321]
	GDF-15	-Induces lipolysis in WA; -Related to stress response.	-Increases energy expenditure; -Potential biomarker for metabolic disorders; -Regulation of appetite.	[118,322]
Osteokines	FGF-23	-Acts on mineral homeostasis; -Action in the regulation of energy metabolism.	-Increased levels in obese people; -Related to fat distribution; -Associated with increased risk of cardiovascular morbidity.	[323,324]
	NGAL	-Metabolic homeostasis; -Acts on immunity; -Cell apoptosis.	-Higher levels in people with Mets; -Suppresses appetite; -Associated with cardiovascular diseases.	[324,325]
	Sclerostin	-Regulates bone formation; -Important in bone resorption; -Inhibits myoblast differentiation.	-Associated with the risk of developing cardiovascular disease; -Higher levels in people with Type 2 diabetes.	[326,327]
	Osteocalcin	-Action on energy homeostasis; -Acts on the bone matrix.	-Increases insulin sensitivity; -Improves glucose tolerance; -Increases energy expenditure.	[328]
	Osteopontin	-Associated with bone calcification; -Acts on inflammation; -Promotes liver inflammation.	-Associated with arterial calcification; -Higher levels in obese people; -Promotes inflammation.	[329,330]
Renokines	Renin	-Activates the RAAS; -Maintenance of sodium homeostasis.	-Strong association of portal hypertension caused by RAAS with obesity; -Increased levels in people with obesity; -Associated with diabetic nephropathy.	[277,331]
	Erythropoietin	-Regulates the production of erythrocytes; -Protects against brain injury; -Acts on the vascular endothelium; -Protects the liver and kidneys against ischemia.	-Improves glucose tolerance; -Increases oxidative metabolism; -Improved insulin sensitivity; -Reduces lipid accumulation in the liver; -May be associated with weight loss.	[332]
	Klotho	-Protective action on the liver; -Regulates phosphate metabolism; -Associated with aging.	-Protects against inflammation; -Antioxidant action; -Low circulating serum levels are associated with obesity; -May improve mitochondrial dysfunction.	[333,334,335]
	NGAL	-Marked associated with kidney injury; -Related to the transport of small hydrophobic molecules.	-Anti-inflammatory action; -Protective action against cellular stress; -Increased levels in people with metabolic disorders.	[336]

Abbreviations: ANGPTL4: angiopoietin-like 4; ANP, atrial natriuretic peptide; BNP, B-type natriuretic peptide; FFA, free fatty acids; FGF-21, fibroblast growth hite 21; FGF-23: Fibroblast Growth Factor 23; GDF-15, growth differentiation factor 15; IL, interleukin; IR, insulin resistance; MED-13, mediator complex subunit 13; MetS, Metabolic syndrome; NGAL, neutrophil gelatinase-associated lipocalin; NO, nitric oxide; ROS, reactive hite species; WAT, hite adipose tissue; BAT, brown adipose tissue.

**Table 2:** Example of the cross-talk of organokines and the role in MetS.

Organokine	Relation with Metabolic Syndrome	Cross-Talk with Other Organokines
Hepatokines	Regulate lipolysis, gluconeogenesis, and hepatic inflammation	Modulate adipokines (leptin) and myokines (FGF-21)
Myokines	Improve insulin sensitivity and browning of adipose tissue	Influence cardiokines (BNP) and hepatokines (FGF-21)
Cardiokines	Control lipolysis, energy expenditure, and cardiac function	Regulate adipokines (adiponectin) and myokines (myostatin)
Adipokines	Modulate inflammation, appetite, and lipid metabolism	Impact hepatokines (resistin) and osteokines (osteocalcin)
Osteokines	Link bone metabolism to glycemic and vascular homeostasis	Interact with rinokines (NGAL) and adipokines (adiponectin)
Rinokines	Regulate blood pressure and kidney damage	Associated with osteokines (FGF-23) and cardiokines (erythropoietin)

## Data Availability

No new data were created or analyzed in this study.
